# Integrating Oral Health into Health Professions School Curricula

**DOI:** 10.1080/10872981.2022.2090308

**Published:** 2022-06-22

**Authors:** Stephanie A. Gill, Rocio B. Quinonez, Mark Deutchman, Charles E. Conklin, Denise Rizzolo, David Rabago, Paul Haidet, Hugh Silk

**Affiliations:** a Family and Community Medicine, Penn State College of Medicine, Hershey, PA, USA; b Office of Academic Affairs, University of North Carolina Adams School of Dentistry, Chapel Hill, NC, USA; c Family Medicine, University of Colorado School of Medicine, Anschutz Medical Campus, Aurora, CO, USA; d Department of Surgery, and Tread Director for Oral Health and Oral Medicine, Virginia Tech Carilion School of Medicine, Roanoke, VA, USA; e Assessment and Evaluation Specialist for the Physician Assistant Education Association, Washington, DC, USA; f Faculty Development, Family and Community Medicine, Penn State College of Medicine, Hershey, PA, USA; g Departments of Medicine, Humanities, and Public Health Sciences, Penn State College of Medicine, Hershey, PA, USA; h Family Medicine and Community Health, UMass Chan Medical School, Worcester, MA, USA

**Keywords:** health professions education, medical education, oral health curriculum, interprofessional education, oral health integration

## Abstract

Oral health is essential to human health. Conditions associated with poor oral health involve all organ systems and many major disease categories including infectious disease, cardiovascular disease, chronic pain, cancer, and mental health. Outcomes are also associated with health equity. Medical education organizations including the Association of American Medical Colleges and National Academy of Medicine recommend that oral health be part of medical education. However, oral health is not traditionally included in many medical school, physician assistant, or nurse practitioner curricula. Several challenges explain this exclusion including lack of time, expertise, and prioritization; we therefore provide suggestions for integrating oral health education into the health professions school curriculum. These recommendations offer guidance for enhancing the oral health curriculum across institutions. We include key organizational and foundational steps, strategies to link oral health with existing content, and approaches to achieve curricular sustainability.

## Introduction

Oral health is an essential part of human health. The World Health Organization defines oral health as ‘a state of being free from chronic mouth and facial pain, oral and throat cancer, oral infection and sores, periodontal (gum) disease, tooth decay, tooth loss, and other diseases and disorders that limit an individual’s capacity in biting, chewing, smiling, speaking, and psychosocial wellbeing’[1]. Oral health is directly connected to overall health outcomes, is associated with health equity, and is an important part of health care across many medical specialties and disciplines[2]. Those with poor oral health experience less effective diabetic control, acute and chronic pain, complications in pregnancy and childbirth, and higher risks of cardiovascular disease, among many other physical conditions[3]. Those with mental health issues and oral disease are more prone to worse anxiety, depression, cognitive status, and severe mental illness[4]. Societal impact is difficult to assess but dental caries is the most common chronic infectious disease in the world[5]. In the USA, more than two-thirds of adults have reported lost work or school hours due to unplanned dental visits, and parents have averaged 2.5 days a year off work due to their children’s dental problems[6]. In the USA, caries is more prevalent among individuals of color and those experiencing poverty, with almost twice as many Black and Mexican American adults having untreated cavities as non-Hispanic White adults[7].

To address the burden of oral disease, health professionals need to learn core concepts of oral health early in training. The amount and type of training across health professions varies greatly. The Association of American Medical Colleges (AAMC) and National Academy of Medicine note that medical education must provide learners with oral health related knowledge, such as risk assessments and exam skills [8–10]. However, in 2012, 91% of U.S. medical school graduates reported that they were either neutral or not well-trained to address oral or dental health topics[11]. In 2018, two-thirds of osteopathic medical schools offered less than six hours of oral health training[12]. Medical schools that incorporate oral health education struggle to achieve competency[13]. While Physician Assistant and Nurse Practitioner Schools have more hours of oral health education, their Deans are still not satisfied with their graduates’ oral health knowledge and skills competency [14,15]. Data are limited, but this gap is understood among health educators to be international in scope[16].

In order to have a comprehensive oral health curriculum in all health professions, best practices should be shared throughout institutions. This will ensure that all health professions are teaching similar content on oral health so that when students enter their professions, they will have received similar competency-based training. This paper draws from the experiences of five U.S. medical schools that routinely teach oral health concepts but can be used in any health professions curriculum. It will share successful incorporation of oral health into the curriculum of the following schools: University of Colorado (CU), University of Massachusetts (UMass), University of North Carolina (UNC), Penn State University (PSU), and Virginia Tech Carilion (VTC). The following review and accompanying tables emerged from thematic analysis of an internal survey, subsequent discussions about the oral health curricula of our five health professions schools, and examination of the references and resource materials we use for teaching oral health.

## Identify and Support an Oral Health Champion

The importance and value of a champion to lead organizational change is well documented in general and for oral health promotion [17,18]. While each of the co-author oral health champions identified the importance of stakeholders and collaborators, each also noted the primacy of a champion. One (HS) noted that even years after curricular implementation, without a champion, the oral health curriculum ‘would all cave in.’ Oral health champions require support from organizational leadership through professional development and small amounts of dedicated time and are best positioned to successfully lead oral health curricular development and integration. Most authors began oral health integration work with some level of external funding or internal protected time, but all noted this work does not require large budgets beyond time. One key to sustaining in-kind faculty support is assuring that oral health teaching time is valued in the promotion process. Additionally, oral health champions can receive peer support through the Society of Teachers of Family Medicine’s Oral Health Collaborative, the American Academy of Pediatrics Section on Oral Health, or the Oral Health Nursing Education Program (OHNEP) (see Table 1). Oral health champions may also benefit from faculty development programs, such as curriculum development courses offered by the Harvard Macy Institute, and formal mentoring relationships.

**Table 1.** Resources and collaborators health schools may find helpful in developing oral health curricula.

Resources or Collaborators	Role/Mission	How to Connect or Find the Resource/Collaborator
American Academy of Pediatrics	Dedicated to the health of all children	National Oral Health Campaign Toolkit: https://www.aap.org/en/news-room/campaigns-and-toolkits/oral-health/ Oral Health Practice Tools: https://www.aap.org/en/patient-care/oral-health/oral-health-practice-tools/ Contact Information for Dental Supply Companies: https://downloads.aap.org/AAP/PDF/fluoride-varnish-manufacturers.pdf AAP Section on Oral Health: https://www.aap.org/en/community/aap-sections/oral-health/ Pennsylvania Healthy Teeth, Healthy Children Program: http://www.healthyteethhealthychildren.org/paaap/healthy-teeth-healthy-children/
American Association of Medical Colleges	MedEdPortal®: An online journal of teaching and learning resources	Dental Collection: https://www.mededportal.org/dental
American Academy of Physician Assistants	Increase Physician Assistant awareness and knowledge of oral health and potential ways to incorporate oral health into PA practice	https://www.aapa.org/cme-central/national-health-priorities/oral-health-initiative/
Area Health Education Centers	Recruiting, training, and retaining a health professions workforce committed to underserved populations	National AHEC Organization Directory: https://www.nationalahec.org/search/custom.asp?id=6189 Health Resources and Services Administration Directory: https://data.hrsa.gov/data/reports/datagrid?gridName=AHECDirectoryReport
Association for Prevention Teaching and Research	Public health learning modules to support health professions workforce education	Public Health Learning Module #15: Oral Health Across the Lifespan: https://www.aptrweb.org/page/PH_LearningModules
Aquifer Virtual Pediatric Patient Cases	Evidence-based, peer-reviewed, and continuously updated virtual cases	Available by subscription: https://aquifer.org/course/
Center for Integration of Primary Care and Oral Health (CIPCOH)	Providing systems-level research on oral health integration into primary care training	https://cipcoh.hsdm.harvard.edu/
Community Dentists	Community-based clinical rotation sites, teaching support	American Dental Association Find-a-Dentist: https://findadentist.ada.org/
Dental Economics	Comparison of fluoride varnishes	Picano L. 2020. Fluoride varnishes: What is the difference, and which one is best? https://www.dentaleconomics.com/science-tech/cosmetic-dentistry-and-whitening/article/14173399/fluoride-varnishes-what-is-the-difference-and-which-one-is-best
Dental Residency Programs	Community-based clinical rotation sites, teaching support	Commission on Dental Accreditation Find a Program: https://coda.ada.org/en/find-a-program
Harvard Macy Institute	Faculty development programs	https://harvardmacy.org/
Health Resources and Services Administration	Advancing oral health and primary care integration	https://bphc.hrsa.gov/qualityimprovement/clinicalquality/oralhealth/index.html
National Maternal and Child Oral Health Resource Center	Offers oral health technical assistance, training, and resources	National Maternal and Child Oral Health Resource Center: https://www.mchoralhealth.org/index.php
Oral Health Coalitions	Promote lifelong oral health through policy, prevention, and education	American Network of Oral Health Coalitions: https://anohc.org/
Oral Health Collaboratives	Peer support and learning on oral health care and integration	Society of Teachers of Family Medicine Oral Health Collaborative: https://connect.stfm.org/communities/allcommunities American Academy of Pediatrics Section on Oral Health: https://www.aap.org/en/community/aap-sections/
Oral Health Nursing Education and Practice (OHNEP)	Tools to help nurse practitioners, nurse-midwives, nurses, and other health professionals incorporate oral health into patient care	http://ohnep.org/
Professional Dental Societies	Supporting dentists, educating the public, and ensuring access to oral health care	American Dental Association and affiliate societies: https://www.ada.org/about/volunteer-and-get-involved-with-the-ada/national-state-local-dental-societies
Smiles for Life National Oral Health Curriculum®	Teaching resources and continuing education modules available from the Society of Teachers of Family Medicine	https://www.smilesforlifeoralhealth.org/
Society of Teachers in Family Medicine	Engaging family physician educators to improve oral health education in family medicine training	https://connect.stfm.org/communities/allcommunities

## Conduct an Oral Health Needs Assessment of the Educational Curriculum, Faculty Experience, and Clinical Environment

Along with having an oral health champion, schools also need to conduct a needs assessment. A needs assessment will allow institutions to better understand the local learning environment that is essential to oral health integration. It should include all aspects of the curricular environment and all relevant stakeholders. The needs assessment should include observations in the various teaching environments, curricular mapping of content, gaps in course content related to oral health, analysis of course descriptions, and interviews, group discussions, and surveys with key informants such as course directors, curriculum leaders, faculty, staff, and learners. One approach to conducting a needs assessment could start with a simple word search in existing curricular documents for the terms ‘oral,’ ‘dental,’ ‘tooth,’ and ‘teeth’ to find what already exists, as CU did. The needs assessment will help identify specific aspects of the overall health professions school curricular infrastructure that can reinforce oral health concepts and encourage students to consider how these would be opportunities to use practical oral health skills. For example, a discussion of the oral microbiome and its impact on systemic disease processes would be appropriate when teaching about the immune system and systemic inflammation. Subsequently, relevant oral health clinical skills concepts can be mirrored during the corresponding clinical skills sessions. A validated tool called the Oral Health Curriculum Evaluation Tool (OHCET) can be used to assign a baseline score and repeated in the future to monitor progress[19].

A needs assessment is also vital to gauge faculty interest and experience in integrating oral health[20]. Because faculty likely have not considered a way – or even need – to integrate oral health into already packed courses, the oral health champion should be prepared to support the importance of oral health in overall health and suggest specific integration ideas. Oral health curricular change can be integrative rather than additive; for example, oral health considerations may be included in a case study, or an oral health article may be used to assess research methodology. Oral health champions can offer to create content to insert into an existing lecture or edit a case that is already in use.

At the same time, it is important to assess the delivery of oral health care in teaching clinics. This way the oral health champion can identify opportunities to bridge content between the preclinical and clinical learning environments and which content to emphasize. For example, PSU first analyzed which oral health diagnoses and billing codes were documented most often by clinicians and in which practice settings (e.g., application of fluoride varnish to young children as part of primary prevention) and then placed students in those settings to observe real-life application of oral health promotion in clinical care.

## Make the Case for Teaching Oral Health Emphasizing Oral-Systemic Health Connections and Health Equity

Many clinicians and health professions educators are not aware of the link between oral health, organ systems, and overall health. Development and later integration of an oral health curriculum begins with making the case for its inclusion. Some in health professions education leadership may not be aware that oral diseases are associated with a growing list of other health problems, including diabetes, cardiovascular disease, arthritis, adverse pregnancy outcomes, and dementia [21–25]. It is important to emphasize these connections and that patients experience significant pain, missed work and school, psychological distress, and costs from oral health conditions[26]. Champions can use local health department statistics and patient stories to reveal the range and importance of oral health topics within and specific to their community. Oral health metrics are also associated with social determinants of health including health equity, racial bias, social justice, and interprofessional teamwork. To discuss oral health access, disparities, and outcomes, health professions educators can use the Oral Health Across the Lifespan module from the Association for Prevention Teaching and Research (Table 1), which provides interprofessional lectures with corresponding classroom activities and discussion guides. The oral health champion can offer to make brief impact presentations at educational leadership meetings or ‘grand rounds’ on the oral-systemic connection, display ready-made curriculum sources such as Smiles for Life (Table 1), and explain how course leaders can integrate small amounts of oral health content into courses to create a school-wide comprehensive curriculum.

Making strategic oral health partnerships can help show leadership that dental providers and educators want to be part of this movement. Many health professions schools may be concerned that they do not have the expertise to teach oral health concepts well. Therefore, a co-located dental school can be an invaluable partner. For health professions schools without a nearby dental school, partnerships with other oral health-related enterprises are possible, including oral health coalitions, community dental providers, dental hygiene schools, community dentists, professional dental societies, dental residencies, dental insurers, dental foundations, local and state public health departments, and other health professions education programs. Strategic partners can provide teaching support, interprofessional education collaboration, experiential learning opportunities, and start-up or ongoing funding as well. See Tables 1 and 2 for examples of collaborators, resources, and potential funders.

**Table 2.** Potential funders for developing oral health curricula.

Potential Funders	Links to Potential Funders
Area Health Education Centers	National AHEC Organization Directory: https://www.nationalahec.org/search/custom.asp?id=6189 Health Resources and Services Administration Directory: https://data.hrsa.gov/data/reports/datagrid?gridName=AHECDirectoryReport
Dental Insurer Foundations	Cigna Foundation: https://www.cigna.com/about-us/corporate-responsibility/cigna-foundation Delta Dental Foundations: multiple foundations exist across U.S. for single states and groups of states The Humana Foundation: https://www.humanafoundation.org/ United Concordia Dental Charitable Fund: https://www.highmarkfoundation.org/about/charitablefunds_ucd.shtml#gsc.tab =0
Health Resources and Services Administration	Search for funding opportunities: https://www.hrsa.gov/grants/find-funding
Oral Health Coalitions	American Network of Oral Health Coalitions: https://anohc.org/
Oral Health Foundations	List of oral health foundations from the National Maternal and Child Oral Health Resource Center: https://www.mchoralhealth.org/links/links_brief.php?wScript=OHFoundationLinks CareQuest Institute for Oral Health: https://www.carequest.org/how-we-work/grantmaking Colgate Bright Smiles, Bright Futures: https://www.colgatepalmolive.com/en-us/community-impact/bright-smiles-bright-futures Crest Toothpaste: https://crest.com/closing-the-smile-gap/
Professional Dental Societies	Academy of General Dentistry Foundation: https://www.agd.org/agd-foundation/our-programs/agd-foundation-grant-program
Regional and Community Foundations	Funding Opportunities for Rural Communities: https://www.ruralhealthinfo.org/topics/oral-health/funding Appalachia Funders Network Health Group: https://www.appalachiafunders.org/health Foundation Directory Online by Candid FDO Quick Start: https://fconline.foundationcenter.org/welcome/quick-start
State and Local Public Health Departments	Centers for Disease Control and Prevention Health Department Directories: https://www.cdc.gov/publichealthgateway/healthdirectories/healthdepartments.html

## Anchor the Curriculum to a Set of Professional Competencies with Clear Knowledge, Attitudes, and Skills Objectives

Competency-based oral health education should prepare students to perform specific professional tasks related to oral health risk assessment and evaluation, preventive interventions, patient communication and education, and interprofessional collaborative practice including proper referrals. The AAMC published oral health competencies for medical students and examples of learning objectives that medical schools can use to develop knowledge, attitudes, and skills related to dental caries, periodontal disease, oral cancer, oral-systemic interactions, and oral public health[8]. The Health Resources and Services Administration (HRSA) identified and described similar competency domains for primary care providers[27]. Likewise, the Center for Integration of Primary Care and Oral Health (CIPCOH) at Harvard University and UMass has recommended a set of oral health integration Entrustable Professional Activities (EPAs) for clinicians entering practice[28]. These EPAs focus on the above competencies as well as practical skills such as referral for common oral health conditions; facilitating patient navigation for oral health care; and working with community partners to identify and prioritize population-based oral health issues. Oral health competencies overlap with other educational competencies as well.

Developing an oral health integration strategy centered on core oral health and crosscutting competencies allows for maximum implementation flexibility. Health professions school leaders and oral health champions can integrate oral health into the courses that can most quickly be modified and with the faculty that are most amenable to incorporating oral health into their teaching. For example, UMass started with an Oral Health Day during third-year clinical clerkships and then more widely integrated oral health into other courses. UNC has focused oral health teaching in its first 18 months of foundation courses. PSU began by aligning oral health with first- and third-year clinical experiences to highlight its relevance to patient care.

## Maximize Oral Health Touchpoints

**Figure 1.**
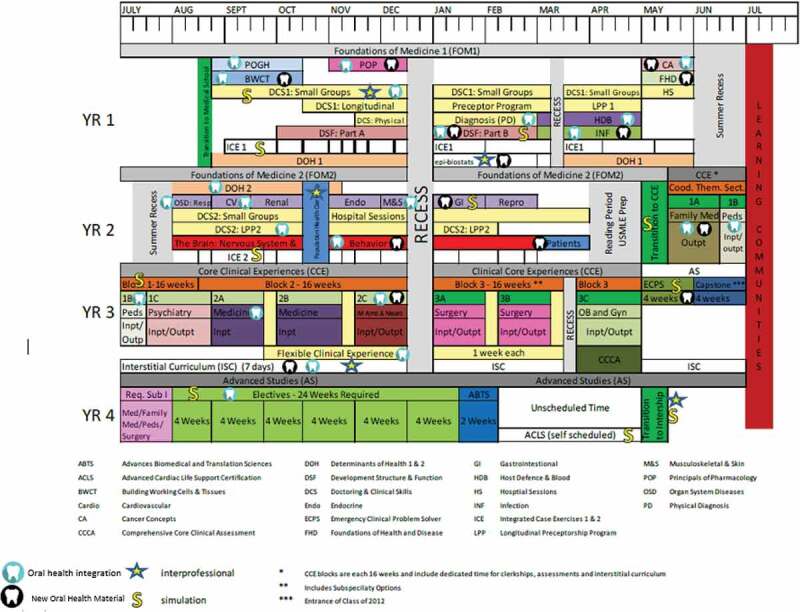
Map of oral health integration into University of Massachusetts Chan Medical School curriculum.

Integrated and spiral curricula allow students to revisit and build on oral health throughout their education. Optimal preclinical courses include organ blocks and those focused on systemic diseases most often related to oral health, including anatomy and physiology, gastroenterology, cardiology, endocrinology, oncology, immunology, reproduction, infectious diseases, genetics, and pharmacology. Oral health assessments, examinations, and fluoride application can be taught in introductory patient interviewing and patient-centered care courses or via hands-on clinical skills workshops. Oral health can be embedded in clinical clerkships or rotations, including family medicine/primary care, pediatrics, emergency medicine, anesthesiology, otolaryngology, and obstetrics and gynecology. Some clerkships use national cases that may already have oral health built into preventive visits (e.g., Aquifer cases in Pediatrics). Oral health electives can provide experiential learning in dental clinics or in handling dental emergencies in a medical setting. The curriculum at UMass, for example, integrates oral health in 18 of 26 preclinical courses and in three pathways for rural, urban, and global health scholars over all four years of medical education including a four-week elective for senior students (Figure 1).

## Pursue Bi-directional Interprofessional Oral Health Education

Oral health should not be taught in silos. Interprofessional oral health education is essential and is at the core of health professional accreditation standards. Health professions schools with affiliated dental schools, such as CU and UNC, have established bi-directional interprofessional education, where dental students and faculty teach medical students how to perform an oral exam and how to apply topical fluoride varnish, while medical students and faculty teach dental students about the manifestations of oral-systemic disease[29]. CU’s Rural Program also includes a skills lab in which dental students train medical students in the performance of oral anesthetic blocks to facilitate orofacial procedures. Other medical schools, such as VTC and UMass, have forged partnerships with local dentists and dental hygiene schools to provide teaching support and experiential learning opportunities for medical students. PSU has coordinated interprofessional maternal and child health case discussions with dental hygiene students from a nearby community college. Several of the authors (RQ, CC, HS) noted the importance and value of interprofessional education to the sustainability and senior leader support of their oral health integration efforts. Furthermore, students enjoy learning with and from colleagues from other disciplines.

## Give Students the Opportunity to Explore Patient Stories to Better Understand the Oral-Systemic Health Connections and Social Determinants of Oral Health

Each author can recall patient experiences that solidified for them the importance of oral health; such personal examples can be powerful learning experiences. Patients can be invited to tell their story during a lecture, faculty can show video clips, or oral health can be integrated into clinical clerkships. Preceptors can encourage students to consider oral health as they gather the patient history and conduct the examination. Oral health champions can also work with clerkship directors to design specific oral health assignments. For example, PSU embedded an oral health project in a required health equity clerkship where students researched oral health resources and conducted and documented a comprehensive oral health visit. At UMass, second year students can explore local oral health resources during a two-week Population Health Clerkship. Activities such as these can highlight the presence and root causes of oral health disparities. Learners can be encouraged to do reflective writing to deepen the patient-based lessons[30].

## Encourage Active Learning and Integrate Oral Health into Clinical Assessments

In recent years, active learning has become a priority among health professions schools. Schools should teach comprehensive oral health examinations and topical fluoride varnish through hands-on workshops. The flipped classroom technique can be employed by asking students to view Smiles for Life online modules prior to hands-on workshops, case study discussions, and team-based learning sessions. Online modules proved invaluable during the early days of the coronavirus pandemic, when in-person teaching and clinical experiences ceased for several months. Smiles for Life has a variety of supplemental materials, such as a question bank and case studies with discussion questions, which can be used for in-class teaching and learning activities. Other active learning resources are listed in Table 1.

Additionally, existing clinical assessments should include an oral health component. Most importantly, the oral examination should be taught changing the ‘HEENT’ exam to ‘HEENOT.’[31] While it is important for students to report improvements in their knowledge, skills, and attitudes related to oral health, it is increasingly important to demonstrate that students consider oral health in their differential diagnoses and consistently assess oral health risk. Clinical assessments should include identification of, attention to, and documentation of oral health concerns, comprehensive oral exam and risk assessment, and oral health-related social determinants of health in standardized and real-life patients.

VTC developed its oral health program and assessment as part of its overall curriculum to create complete integration. By comparison, the other schools added oral health topics to an existing curriculum. Not surprisingly ‘retrofitting’ can be significantly more difficult regarding all aspects of curricular integration and assessment. PSU’s oral health champion successfully worked for two years to integrate oral health into the existing first year medical student objective structured clinical examinations (OSCE). All oral health offerings should include some form of assessment including pre/post multiple choice questions, short answers, or chart reviews.

At a clinical level, it is important to train clinical faculty and health professions students to practice what the school is teaching. This often will mean workflow adaptation, which can be done through quality improvement projects and monitoring billing data. In pediatric departments, oral health quality improvement has been robust and even linked to board recertification for faculty[32]. PSU planned for faculty development and clinical quality improvement from the beginning, with an initial focus on application of topical fluoride varnish in children under age six. The Center for Integration of Primary Care and Oral Health (CIPCOH) trains state oral health education champions to work with health professions school faculty to improve their oral health curricula.

## Refine, Innovate, and Adapt the Curriculum for Sustainability

Essential to all enduring curricula is the need to anticipate and adapt in response to changing healthcare needs and constraints creatively and nimbly. UMass is currently revamping its oral health curriculum as the school undergoes an overall curriculum renewal; CU has refined and adapted its oral health offerings several times; and PSU already modified much of its oral health curriculum. We anticipate ongoing changes as our understanding of oral health in the COVID era improves. Learner feedback is essential in this process. The result is a continuous process of oral health curricular assessment, refinement, and adaptation to align our teaching with emerging oral health data; practices to facilitate oral health integration and routine health care; and new educational strategies to better support comprehension and retention of oral health concepts. In order to ensure that an oral health curriculum and its integration outlasts the champion’s tenure, other faculty must be recruited and developed. New faculty can observe the champion or a dental colleague teaching, and then be observed leading the lesson at the next opportunity from the experienced faculty member and be provided with feedback.

## Conclusion

Successful development and integration of oral health into a health professions school curriculum is challenging and multifaceted. Given the health inequities associated with oral health, health care professionals engaged in clinical care should have the desire to teach this important topic. The requisite skills and knowledge to conduct oral health assessments, offer preventive advice, and start initial management including dental referrals can easily be enhanced or taught to faculty. Furthermore, embedding oral health into the health professions school curriculum will strengthen interprofessional care between the medical and dental communities. We encourage champions to share their oral health curricular innovations and outcomes through conference presentations and peer-reviewed publication. By increasing our collective knowledge and voice about oral health in health education, we can teach our learners more effectively about oral health, deliver more complete care, and ultimately provide our patients with better, more equitable care.

## Biographical Notes for Each Author

Stephanie A. Gill, MD, MPH, is an Assistant Professor of Family and Community Medicine at the Penn State College of Medicine. She is leading a five-year, federally funded grant to integrate oral health and primary care.

Rocio B. Quinonez, DMD, MS, MPH, is Associate Dean for Curriculum, and Professor at the University of North Carolina Adams School of Dentistry in Chapel Hill.

Mark Deutchman, MD, is Professor of Family Medicine at the University of Colorado School of Medicine’s Anschutz Medical Campus. He is Associate Dean for Rural Health.

Charles E. (Bud) Conklin, DDS, is Associate Professor of Surgery and Tread Director for Oral Health and Oral Medicine at Virginia Tech Carilion School of Medicine.

Denise Rizzolo, PhD, MPH, PA-C, is an Assessment and Evaluation Specialist for the Physician Assistant Education Association, Washington, DC

David Rabago, MD, is Professor and Vice Chair for Faculty Development in the Department of Family and Community Medicine at Penn State College of Medicine.

Paul Haidet, MD, MPH, is Distinguished Professor in the Departments of Medicine, Humanities, and Public Health Sciences at the Penn State College of Medicine.

Hugh Silk, MD, MPH, is Professor of Family Medicine and Community Health at the UMass Chan Medical School. He also is a Principal Investigator at the Center for Integration of Primary Care and Oral Health (CIPCOH) at Harvard University.
